# ProQ prevents mRNA degradation through inhibition of poly(A) polymerase

**DOI:** 10.1093/nar/gkaf103

**Published:** 2025-02-27

**Authors:** Sofia Bergman, Christopher Birk, Erik Holmqvist

**Affiliations:** Department of Cell and Molecular Biology, Biomedical Centre, Uppsala University, 75124 Uppsala, Sweden; Department of Cell and Molecular Biology, Biomedical Centre, Uppsala University, 75124 Uppsala, Sweden

## Abstract

The RNA-binding protein ProQ interacts with many transcripts in the bacterial cell. ProQ binding is associated with increased messenger RNA (mRNA) levels, but a mechanistic explanation for this effect has been lacking. In *Salmonella* Typhimurium, ProQ affects key traits associated with infection, including motility and intracellular survival. However, the direct links between ProQ activity and these phenotypes are not well understood. Here, we demonstrate that ProQ promotes biofilm formation, another virulence-associated phenotype. This effect is strictly dependent on sigma factor RpoS. ProQ increases both RpoS protein and *rpoS* mRNA levels, but neither affects *rpoS* transcription nor translation. The *rpoS* mRNA is a ProQ target, and expression of the *rpoS* 3′UTR alone is strongly dependent on ProQ. RpoS expression becomes independent of ProQ in strains lacking poly(A) polymerase I (PAPI), indicating that ProQ protects against 3′ end-dependent decay. Indeed, purified ProQ inhibits PAPI-mediated polyadenylation at RNA 3′ ends. Finally, PAPI is required for ProQ’s effect on expression of genes involved in biofilm, motility, osmotic stress, and virulence, indicating that inhibition of polyadenylation is a general function of ProQ.

## Graphical Abstract

**Graphical Abstract:**
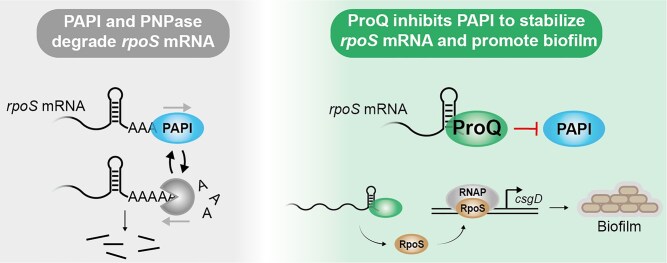

## Introduction

RNA-binding proteins of the ProQ/FinO family are ubiquitously found in alpha-, beta-, and gammaproteobacteria [1]. Members of this protein family share the ProQ/FinO-domain, which was first discovered and characterized in the fertility inhibition protein (FinO) of F plasmids [2]. While FinO binds a single RNA target, chromosomally encoded proteins of this family, such as ProQ in *Enterobacteriaceae, Pasteurellaceae*, and *Neisseriaceae*, bind hundreds of different transcripts [3–6, 7]. Despite these differences in target scope, *all* characterized ProQ/FinO family proteins preferentially bind terminator stem-loop structures at RNA 3′ ends. The structural basis for RNA binding by the ProQ/FinO-domain was recently revealed in a crystal structure of the *Legionella* RocC protein in complex with the transcription terminator of its main small RNA (sRNA) target RocR. In this structure, the RNA 3′ end is recognized by a highly conserved pocket in the ProQ/FinO domain, while the 3′ side of the terminator stem interacts with an alpha-helical N-cap structure reminiscent of the mammalian Roquin protein [8]. These results align well with biochemical analyses of RNA recognition determinants of *Escherichia coli* (*E. coli*) ProQ [9, 10], indicating a conserved mechanism of 3′ end binding by the ProQ/FinO domain.

A common function among ProQ/FinO proteins is their ability to stabilize RNA ligands. For instance, ProQ in *Salmonella enterica*serovar Typhimurium (*S*.Tm) and *Neisseria meningitidis* protects messenger RNA (mRNA) targets from exoribonucleolytic degradation [5, 7], and global RNA stability measurements in *S*.Tm estimated that ProQ affects the stability of one-third of its RNA targets [11]. In these bacteria, degradation from the 3′ end is carried out by three major enzymes; RNase R, RNase II, and PNPase [12]. Their efficiency is dependent on 3′ end accessibility, which is strongly stimulated by polyadenylation by poly(A) polymerase I (PAPI) [13]. However, it has been unclear whether ProQ directly interferes with the activity of any of these enzymes.

Though molecular mechanisms of ProQ-dependent RNA stabilization remain unclear, the phenotypical consequences of the resulting altered gene expression are well described. Deletion of *proQ* in *S*.Tm leads to reduced invasion of, and intracellular survival in, mammalian host cells [14]. This is not surprising considering that ProQ is required for proper expression of both the flagellar pathway and the intracellular virulence expression program [14, 15]. ProQ also promotes survival in the presence of high concentration of antibiotics [16] and during oxidative stress [11], and promotes biofilm formation in several bacterial species [17–20].

In this study, we investigated the mechanistic basis of ProQ’s role for biofilm formation in *S*.Tm. Our results indicate that ProQ increases extracellular matrix production by promoting the expression of RpoS, an alternative sigma factor required for proper biofilm formation. This is achieved through an interaction between ProQ and the 3′ end of *rpoS* mRNA, leading to increased mRNA levels. In the absence of PAPI, ProQ becomes dispensable for RpoS expression. *In vitro*, ProQ binding to RNA 3′ ends inhibits PAPI-dependent polyadenylation, providing a mechanistic explanation for RNA stabilization by ProQ/FinO proteins. *In vivo*, the absence of PAPI abolishes ProQ-dependent activation not only of biofilm genes, but also genes within the flagellar, virulence, and oxidative stress pathways. This suggests that ProQ-dependent inhibition of PAPI-mediated polyadenylation is a general mechanism.

## Materials and methods

### Bacterial strains and growth conditions

Bacterial strains used in this study are listed in Supplementary Table S1. Gene deletions were generated by P22-mediated transduction from previously constructed mutant strains into the *S.Tm* wild-type strain SL1344 (Supplementary Table S1). Antibiotic cassettes on the chromosome were removed using the pCP20 plasmid [21]. Bacterial cultures were grown in Lysogeny Broth (LB) medium at 37°C or 28°C shaking at 220 rpm. To mimic intracellular conditions, a minimal medium with low pH and low Mg^2+^ concentration was used (here called SPI2 medium), as previously described [22]. Antibiotics (ampicillin 100 μg/ml, chloramphenicol 30 μg/ml, tetracycline 12.5 μg/ml, and/or kanamycin 50 μg/ml) were supplemented when appropriate. Isopropanyl ß-D-1-thiogalactopyranoside (IPTG) was added to the media at a final concentration of 0.5 mM to induce expression of ProQ from a plasmid. Arabinose (0.02%) was added for 30 min to induce expression of the 3′ *rpoS* fragment.

### Plasmid construction

Oligonucleotides and plasmids are listed in Supplementary Tables S2 and S3, respectively. The transcriptional fusions of *rpoS* (pSB013) and *csgD* (pSB017) were constructed by cloning the respective promoter sequences (primers EHO-1902/-1903 and EHO-2328/-2329, respectively) between *Bam*HI and *Xho*I sites in plasmid pUA66. The translational *rpoS-gfp* fusion (pSOB001) was constructed by inserting a fragment from the *rpoS* transcription start site to the 20th codon of the *rpoS* ORF (primers EHO-1884/1885) between *Ns*iI and *Nhe*I sites in plasmid pXG10-SF. All reporter gene fusion plasmids constructued in this study are schematically described in Supplementary Fig. S1. Plasmid pSB015, allowing arabinose-inducible expression of the *rpoS* 3′ fragment, was constructed by cloning a polymerase chain reaction (PCR) product (primers EHO-1945/-1946) between *Nhe*I and *Hin*dIII in plasmid pEH809 [15].

### RNA extraction and Northern blot

Total RNA was extracted by the hot phenol method as in [5]. RNA samples (8–10 μg) were diluted 1:1 in RNA loading buffer (0.025% w/v xylene cyanol, 0.025% w/v bromophenol blue in formamide), denatured at 95°C, and loaded on 6% (v/v) polyacrylamide (PAA)/8 M urea gels together with a radiolabeled pUC19 MSP1 marker (Thermo Fischer). RNA was transferred to a nitrocellulose Hybond-N+ membrane (Amersham, Cytiva) by wet electroblotting at 4°C for 2 h at 360 mA, crosslinked at 1200 mJ ultraviolet light, and prehybridized in Church buffer [0.25 M phosphate buffer, pH 7.2, 1 mM ethylenediaminetetraacetic acid solution (EDTA), 7% sodium dodecyl sulfate (SDS)] [23] for 45 min at 42°C. Subsequently, a 5′-^32^P-labeled DNA oligonucleotide was added to the hybridization buffer, and incubation continued for 2–24 h. Membranes were washed three times in 0.5 × saline-sodium citrate/0.1% SDS. Dried membranes were exposed to a phosphor screen, and radioactive signals detected using a Typhoon phosphorimager (Cytiva).

### Quantitative reverse transcription PCR (qRT-PCR)

Total RNA samples were DNase-treated with TurboDNase for 15 min at 37°C, and the enzyme was subsequently inactivated with 15 mM EDTA at 75°C for 10 min. Two micrograms of RNA was converted to complementary DNA (cDNA) using Maxima H Minus First Strand cDNA Synthesis Kit (#K1652, Thermo Scientific). Power SYBR Green PCR Master Mix and cDNA, equivalent to 40 ng RNA, was mixed and analyzed with a Step one Plus real time PCR system (Applied Biosystems). The levels of *rpoS* mRNA were determined using primers EHO-1958/1958, and transfer-messenger RNA (tmRNA) (EHO-2368/2369) levels were used for normalization.

### Western blot

Bacteria were grown in LB to an OD_600_ of 2 and pelleted by centrifugation. Pellets were resuspended in Laemmli Sample Buffer (Bio-Rad), denatured at 95°C, separated on Mini-PROTEAN TGX Stain-Free Protein Gels (Bio-Rad), and transferred to a polyvinylidene difluoride membrane using the Trans-Blot Turbo Transfer Starter System (Bio-Rad). The membrane was blocked with 3% bovine serum albumin (BSA) (Sigma) in tris-buffered saline with Tween 20 (TBS-T) buffer overnight at 4°C, washed with TBS-T, and probed with an anti-GroEL-Peroxidase Conjugate antibody produced in rabbit 1:50 000 (Sigma) or anti-RpoS antibody at a 1:1000 (BioLegend) in 3% BSA TBS-T for 1 h at room temperature (r.t.). The anti-RpoS antibody was followed by incubation with an horseradish peroxidase-conjugated anti-mouse antibody at 1:100 000 (Sigma) in TBS-T 3% BSA for 1 h. The membrane was then developed with enhanced chemiluminescence detection reagent (Amersham, Cytiva) and scanned in a ChemiDoc MP System (Bio-Rad).

### Florescence measurements of promoter fusions

Overnight bacterial cultures were diluted 1:100 in LB or SPI2 medium, loaded in a 96-well plate, and grown for 20 h at 37°C or 28°C, shaking for 30 sec every 10 min in a plate reader (Tecan infinite 200Pro). Florescence signals (GFP, BFP, and YFP) and optical density (OD_600_) were measured every 10 min.

### Biofilm assays

To monitor biofilm formation on solid media, bacteria were grown in LB overnight at 37°C, and 10 μl aliquots of overnight cultures were spotted on low salt Lysogeny Broth agar (LA) plates (1 mM NaOH) containing Congo red (40 μg/ml), brilliant blue (20 μg/ml), and tetracycline (12.5 μg/ml). The plates were incubated at 28°C for 8 days and then photographed. Analysis of biofilm formation in liquid media was performed as previously described [24] with a few modifications. Overnight cultures were diluted 1:200 in low salt LB (1 mM NaOH) in glass tubes and incubated without aeration at 28°C. Biofilm development and adherence to the wall of the glass tubes were followed over time. After 8 days the liquid was carefully removed and the tubes were air dried and heat fixed at 60°C for 1 h. The tubes were incubated at r.t with 100% methanol rotating for 15 min, followed by a rotating incubation with a crystal violet solution (1% crystal violet in 50% methanol) for 10 min. After rinsing with deionized water and air drying, biofilm formation was quantified by dissolving adhered material in 30% acetic acid and measuring optical density at 570 nm.

### Electrophoretic mobility shift assay

RNA was *in vitro* transcribed with MEGAscript T7 transcription kit (Life Technologies) from annealed oligonucleotides (see Supplementary Table S2) and labeled with ^32^P at the 5′ end; 2.2 nM of labeled RNA was mixed with increasing concentrations of purified ProQ [5] in reaction buffer (25 mM TrisHCl, pH 7.4, 100 mM NaCl, 1 mM MgCl_2_) for 5 min at 37°C. After incubation, samples were separated on native polyacrylamide [6% PAA, 0.5× Tris-borate-EDTA (TBE) buffer] gels at 4 °C, and radioactive signals were detected using a Typhoon phosphorimager (Cytiva).

### Polyadenylation assay

*In vitro* transcribed and ^32^P-labeled RNA (MEGAscript) was denatured, mixed in a high magnesium buffer (25 mM Tris–HCl, pH 7.4, 100 mM NaCl, 10 mM MgCl_2_), and incubated for 5 min at 37°C. The RNA mix (including 2.5 nM of RNA) was then incubated for 20 min at 37°C with increasing concentrations of purified ProQ. Subsequently, 0.25 U of *E. coli* poly(A) polymerase (New England BioLabs) and ATP (final concentration 1 mM) was added, and the mix incubated for 20 min at 37°C. Samples were mixed with quenching buffer (0.025% xylene cyanol, 0.025% bromophenol blue, 20 mM EDTA in formamide), denatured, and separated on 6% (v/v) PAA/8 M urea gels alongside a radiolabeled pUC19 MSP1 marker. Radioactive signals were detected using a Typhoon phosphorimager (Cytiva).

## Results

### ProQ is required for biofilm formation in S.Tm

**Figure 1.**
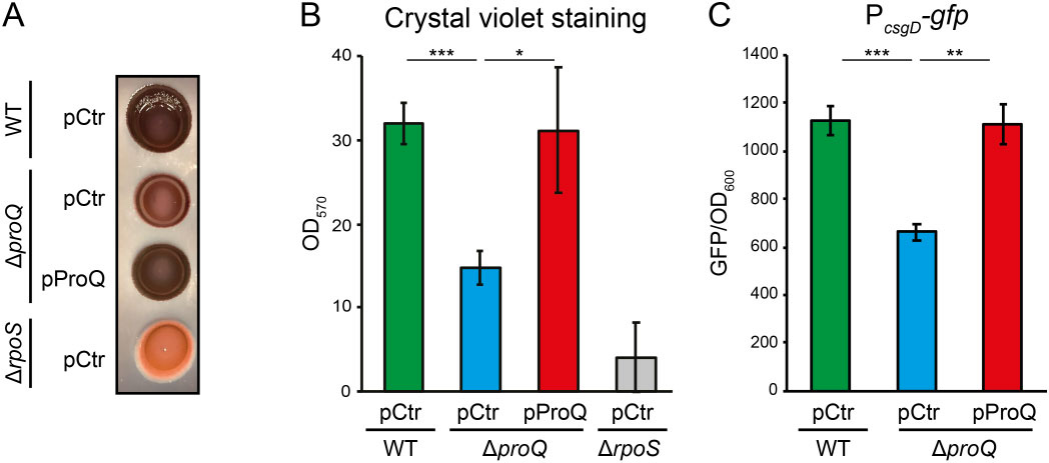
ProQ promotes biofilm formation in *S*.Tm. (**A**) Colony morphology during growth on Congo red agar plates. Indicated strains were grown for 8 days at 28°C. (**B**) Biofilm formation in static liquid cultures. Indicated strains were grown in low salt LB for 8 days at 28°C. The adherent biofilm was stained with crystal violet and quantified by measuring OD_570_. (**C**) Measurements of GFP from the transcriptional reporter P*_csgD_*-*gfp* during growth in LB at 28°C. ProQ expression from the pProQ plasmid is comparable to endogenous ProQ levels. pCtr is the parental plasmid for pProQ and served as an empty vector control. Bars [panels (B) and (C)] show mean values from three biological replicates. Error bars denote standard deviation. Statistical difference was determined using a two-tailed *t*-test (*P < .1; **P < .05; ***P < .01).

Previous studies have shown that deletion of *proQ* leads to reduced biofilm formation in various bacterial species [17–19, 25]. To test whether ProQ affects biofilm formation also in *S*.Tm, we monitored colony morphology during growth on Congo red agar plates. The wild-type strain showed the expected rdar (red, dry, and rough) morphotype resulting from dye binding to the extracellular matrix components curli and cellulose [26], while the non-biofilm producing Δ*rpoS* strain showed a smooth and white morphotype (Fig. 1A). The *proQ* deletion strain formed light red colonies, indicative of reduced matrix production, whereas a Δ*proQ* strain harboring plasmid pProQ phenocopied the wild-type strain (Fig. 1A). Note that pProQ expresses ProQ from an inducible promoter which, in the absence of inducer, provides leaky ProQ expression comparable to endogenous ProQ levels [27]. To further examine ProQ’s effect on biofilm formation, static liquid cultures were subjected to crystal violet staining. Consistent with the biofilm assay on plates, the Δ*proQ* strain showed reduced crystal violet staining compared to the wild-type and the ProQ complementation strain (Fig. 1B). Production of curli and cellulose in *S*.Tm is dependent on the master transcription factor CsgD. To test whether the ProQ-dependent reduction in biofilm formation was due to reduced *csgD* expression, we monitored expression from a transcriptional P*_csgD_-gfp* reporter. Deletion of *proQ* resulted in significantly reduced expression compared to the wild-type strain or the ProQ complementation strain (Fig. 1C). Thus, ProQ is required for proper transcription of *csgD*, and for biofilm formation. Since ProQ acts at the post-transcriptional level, the ProQ-dependent effect on *csgD* transcription initiation should be indirect, and could stem from a direct effect on expression of one of the many regulators known to act at the *csgD* promoter.

### ProQ activates *csgD* transcription through RpoS

**Figure 2.**
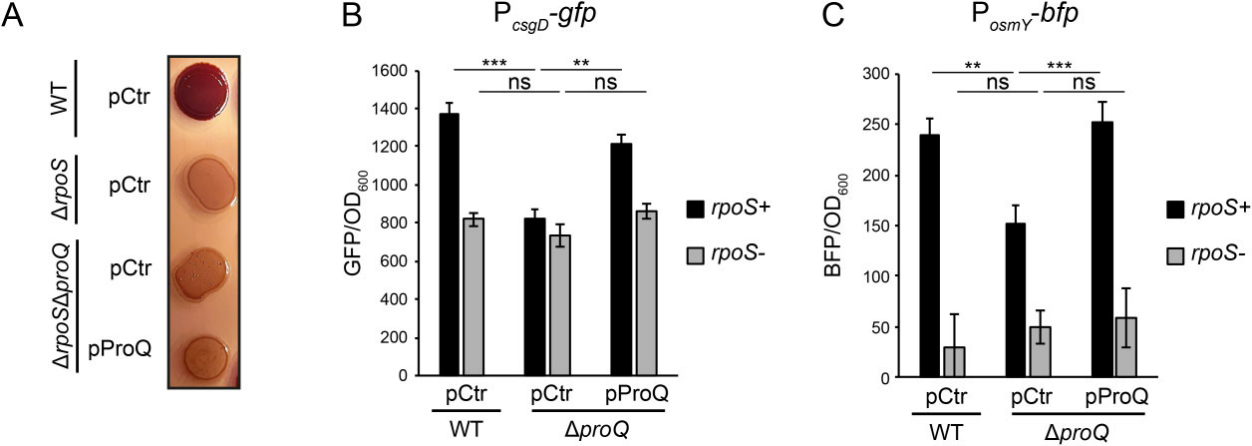
The effect of ProQ on biofilm and *csgD* transcription is RpoS-dependent. (**A**) Colony morphology of indicated strains during growth at 28°C on Congo red agar plates. Measurements of GFP from the transcriptional reporter P*_csgD_*-*gfp* (**B**) or BFP from P*_osmY_*-*bfp* (**C**) in the presence or absence of RpoS and/or ProQ. Indicated strains were grown in LB at 28°C. Bars show mean values from three biological replicates. Error bars denote standard deviation. Statistical difference was determined using a two-tailed *t*-test (*P < .1; **P < .05; ***P < .01).

The *csgD* promoter is a hub for many different transcription factors, two-component systems, and sigma factors. To find a factor that could link ProQ to reduced expression of *csgD*, we searched our previous CLIP-seq data sets [5] for ProQ-bound mRNAs encoding known *csgD* regulators. This singled out *rpoS*, encoding the stress sigma factor RpoS, as a plausible candidate. In a strain lacking *rpoS*, colony morphology on Congo red plates were unaffected by deletion or complementation of *proQ* (Fig. 2A), suggesting that ProQ’s effect on biofilm formation is RpoS-dependent. To test whether ProQ-dependent activation of *csgD* transcription requires RpoS, we monitored expression from the *csgD* promoter in an *rpoS* deletion strain. In the absence of RpoS, the rate of *csgD* transcription was reduced but not abolished (Fig. 2B). This is expected since *csgD* is not only transcribed by RNA polymerases carrying RpoS, but also those associated with the housekeeping sigma factor RpoD [28]. Deletion of *proQ* in the *rpoS* background did not further decrease *csgD* transcription (Fig. 2B), indicating that ProQ-dependent activation of *csgD* transcription requires RpoS. Moreover, since the remaining RpoD-dependent expression from P*_csgD_* was independent of ProQ, this protein does not have a general effect on transcription, as demonstrated using a synthetic RpoD-dependent promoter (Supplementary Fig. S2). In addition to biofilm, RpoS controls many other stress-related pathways. We therefore monitored transcription from another RpoS-dependent promoter, P*_osmY_*, which drives expression of an osmotic stress-induced protein. Deletion of *proQ* resulted in significantly reduced activity of the P*_osmY_* promoter compared to the wild-type or complementation strains (Fig. 2C). This effect was abolished in a strain lacking RpoS (Fig. 2C), suggesting a general effect of ProQ on RpoS-dependent promoters.

### ProQ promotes expression of RpoS

**Figure 3.**
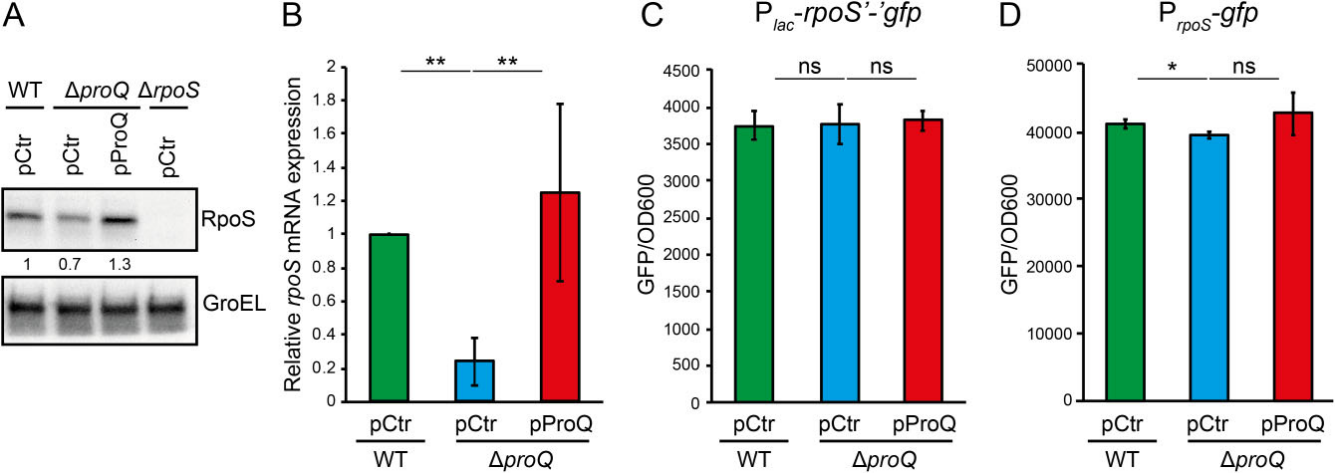
ProQ positively affects expression of RpoS. (**A**) Protein levels of RpoS monitored by western blotting using an anti-RpoS antibody. Indicated strains were grown in LB at 37°C to an OD_600_ of 2.0. GroEL served as loading control. Values below the RpoS blot denote RpoS band intensities normalized to GroEL. Wild-type strain values were set to unity. (**B**) Steady-state levels of *rpoS* mRNA determined by RT-qPCR for the indicated strains grown in LB at 37°C to an OD_600_ of 2.0. Measurements of GFP from the translational fusion P*_lac_*-*rpoS’-‘gfp* (**C**) and the transcriptional fusion P*_rpoS_-gfp* (**D**) during growth in LB at 37°C. Bars [panels (**B–D**) show mean values from three biological replicates. Error bars denote standard deviation. Statistical difference was determined using a two-tailed *t*-test (*P < .1; **P < .05; ***P < .01).

Next, we investigated whether expression of RpoS itself is controlled by ProQ. Western blot analysis showed that deletion of *proQ* is accompanied by reduced RpoS protein levels (Fig. 3A), and complementation of ProQ *in trans* promoted RpoS expression beyond wild-type levels (Fig. 3A). Deletion of *proQ* also resulted in significantly reduced *rpoS* mRNA levels, as determined by qRT-PCR. Again, ProQ complementation restored *rpoS* mRNA expression to wild-type levels (Fig. 3B). In principle, the effect of ProQ on *rpoS* expression could stem from perturbations of transcription, translation, or mRNA stability. To test this, we monitored expression of both a transcriptional P*_rpoS_-gfp* fusion, and a translational *rpoS’-‘gfp* fusion expressed from a heterologous promoter. Both *rpoS* transcription and translation were completely unaffected by deletion or complementation of *proQ* (Fig. 3C and D).

### ProQ acts at the *rpoS* 3′ end

**Figure 4.**
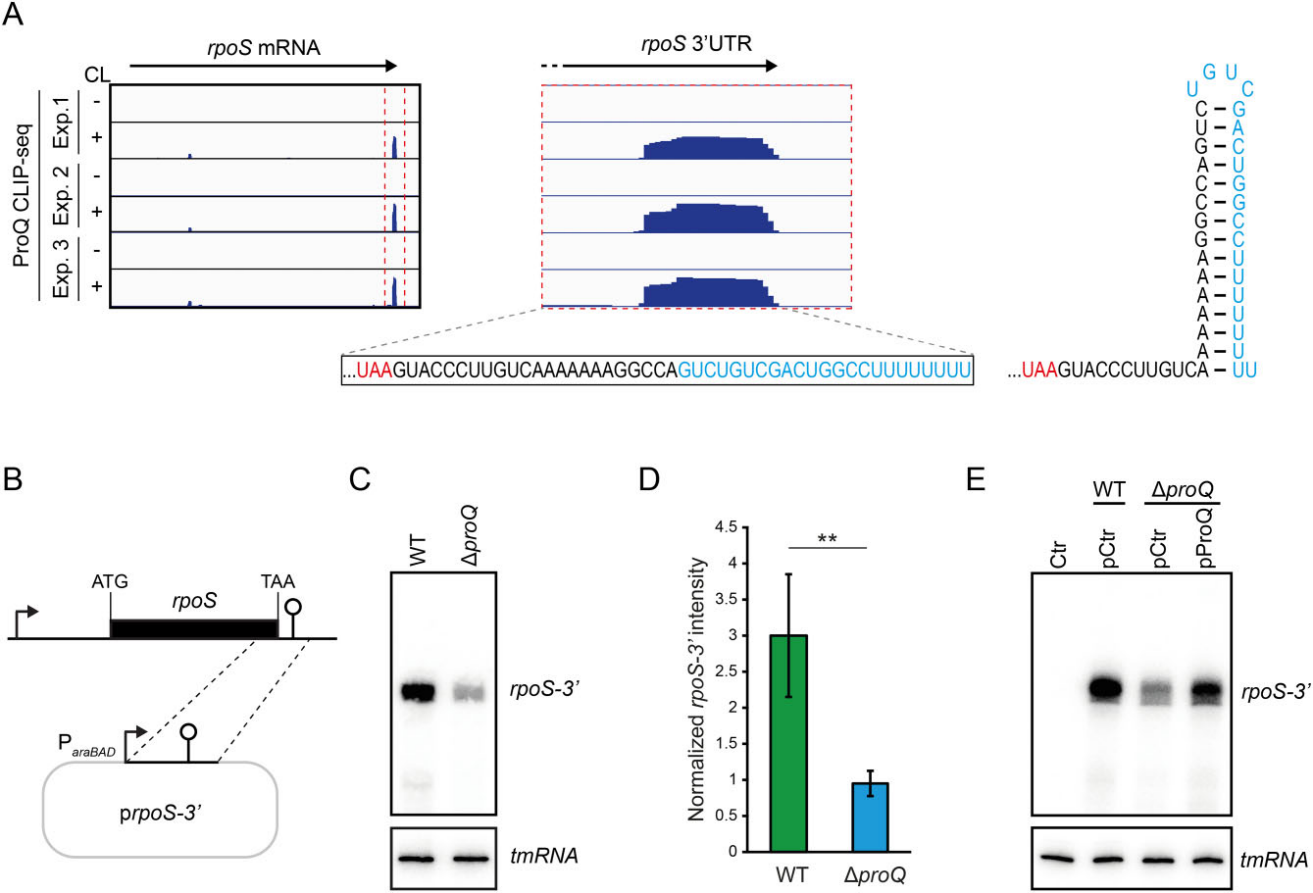
ProQ acts at the 3′ end of *rpoS* mRNA. (**A**) Read coverage from ProQ CLIP-seq in *S*.Tm grown in LB to early stationary phase [5]. The enlarged view shows read coverage at the ProQ binding site in the *rpoS* 3′UTR. Below is the sequence and to the right the predicted secondary structure (RNAfold webserver [29]) of the ProQ binding site at the *rpoS* terminator. CL, crosslinking. (**B**) Schematic overview of the p*rpoS-3′* plasmid (pSB015) expressing a 3′ fragment of the *rpoS* mRNA. (**C**) Northern blot analysis of the *rpoS* 3′ fragment expressed in wild-type and Δ*proQ* strains. Probing of tmRNA serves as loading control. (**D**) Quantification of three independent Northern blot experiments as shown in panel (C). Bars show mean values of *rpoS-3′* band intensities normalized to tmRNA. Error bars denote standard devation. **P < .05. (**E**) Northern blot analysis of *rpoS-3′* expression in a wild-type, Δ*proQ*, and ProQ complementation strain. In panels (C) and (D), the indicated strains were grown in LB at 37°C to mid-exponential phase, after which arabinose was added at a final concentration of 0.02% for 30 min to induce expression of the *rpoS-3′* fragment. A 5′ labeled DNA oligonucleotide specific for the *rpoS* 3′ end was used for detection. tmRNA served as loading control.

The fact that ProQ promoted *rpoS* mRNA levels, but neither transcription nor translation, indicated that ProQ may protect the *rpoS* mRNA from degradation. Revisiting our previous CLIP-seq data [5] in more detail showed that the 3′ end of *rpoS* mRNA harbors a ProQ binding site starting from the loop of the terminator hairpin and extending to the very end of the transcript (Fig. 4A). To test whether the effect ProQ exerts on *rpoS* is through the 3′ end of the mRNA, and indeed independent on native *rpoS* transcription and translation, we constructed a plasmid that, from the heterologous P*_araBAD_* promoter, expresses a transcript starting 100 nucleotides upstream of the 3′ end of the *rpoS* mRNA (Fig. 4B). Northern blot analysis showed that expression of the *rpoS-3′* transcript was reduced in the Δ*proQ* strain compared to the wild-type strain (Fig. 4C). Quantification of three independent experiments verified that this reduction was statistically significant (Fig. 4D). In line with this, the *rpoS-3′* transcript was destabilized upon *proQ* deletion (Supplementary Fig. S3). Complementing the *proQ* deletion with expression of ProQ *in trans* fully restored *rpoS-3′* levels (Fig. 4E).

### ProQ’s effect on *rpoS* expression depends on PAPI

**Figure 5.**
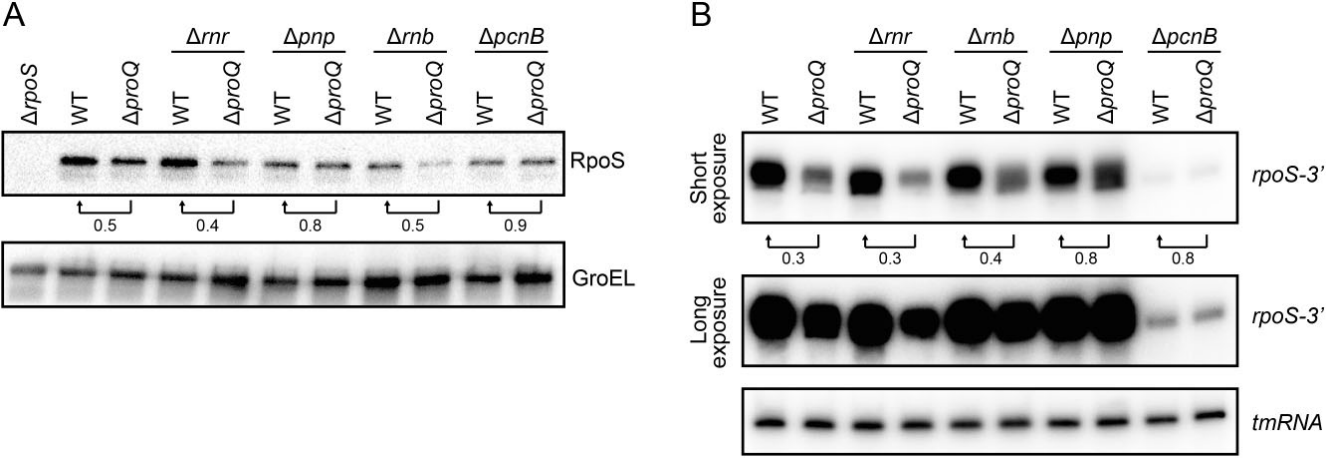
ProQ’s effect on RpoS levels is PAPI-dependent. (**A**) Western blot monitoring RpoS levels in the indicated strains grown in LB at 37°C to an OD_600_ of 2.0. RpoS levels were detected with an anti-RpoS antibody and GroEL served as loading control. Numbers below the RpoS bands indicate the relative reduction in RpoS levels upon deletion of *proQ* in the indicated strain backgrounds. RpoS levels were normalized to GroEL. (**B**) Northern blot analysis of the *rpoS* 3′ fragment expressed from p*rpoS-3′* (Fig. 4) in the indicated strains grown in LB at 37°C after induction with 0.02% arabinose for 30 min. A 5′ labeled DNA oligonucleotide specific for the *rpoS* 3′ end was used for detection. tmRNA served as loading control. Numbers below the *rpoS-3′* bands indicate the relative reduction in *rpoS-3′* levels upon deletion of *proQ* in the indicated strain backgrounds. The *rpoS-3′* levels were normalized to tmRNA.

Since ProQ binds the 3′ end of *rpoS* mRNA and enhances *rpoS* mRNA levels (Figs 3 and 4), protection against a 3′-dependent degradation activity seemed to be a likely scenario. To test this, we monitored RpoS protein levels in strains lacking any of the three major 3′ to 5′-dependent exoribonucleases: RNase R, RNase II, and PNPase, encoded by genes *rnr*, *rnb*, and *pnp*, respectively. We also included a deletion of *pcnB*, encoding poly(A) polymerase PAPI, which stimulates exoribonucleolytic activity by providing extended single-stranded 3′ ends. Western blot analysis showed that the reduction in RpoS levels observed in the *proQ* deletion strain remained in strains lacking either RNase R or RNase II (Fig. 5A). However, in the absence of either PNPase or PAPI, the RpoS levels became insensitive to deletion of *proQ* (Fig. 5A). In line with this, Northern blot analysis showed that deletion of *proQ* did not affect the levels of the *rpoS-3′* transcript in the absence of PNPase (Fig. 5B). In the *pcnB* deletion strain, *rpoS* 3′ transcript levels were strongly reduced compared to wild-type (Fig. 5B). This is due to the strongly reduced copy number of the ColE1-type plasmid encoding the *rpoS 3′* fragment (Supplementary Fig. S4); the repressor of replication, RNA I, is stabilized in *pcnB* deletion backgrounds [30, 31]. Importantly, deletion of *proQ* in the Δ*pcnB* background did not further reduce *rpoS* 3′ expression, indicating that PAPI is required for ProQ-dependent stabilization. Together, this indicates that ProQ protects against PAPI- and PNPase-dependent degradation of the *rpoS* mRNA.

### ProQ counteracts PAPI-dependent polyadenylation at RNA 3′ ends

**Figure 6.**
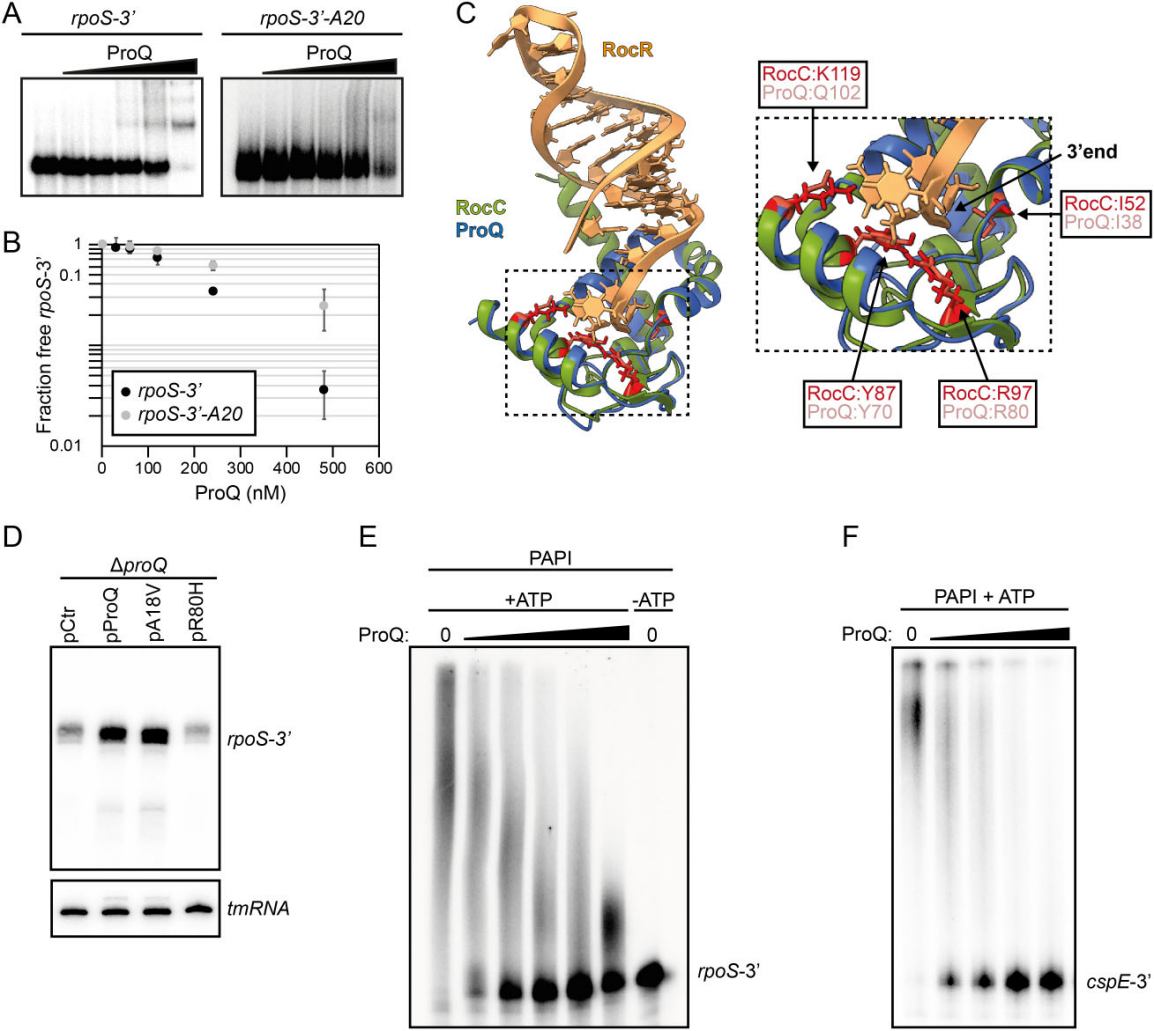
ProQ inhibits PAPI-dependent polyadenylation. (**A**) Electrophoretic mobility shift assay (EMSA) of binding between purified ProQ and *in vitro* transcribed and radioactively labeled *rpoS* 3′, with or without a 20 nucleotide poly(A)-tail. (**B**) Quantification of three independent EMSAs as shown in panel (A). Points denote mean values. Error bars show standard deviations. (**C**) Left: Alignment of the AlphaFold model of the ProQ/FinO domain of *S*.Tm ProQ and the crystal structure of the RocC–RocR complex (PDB ID: 7RGU). RocC residues involved in RocR 3′ recognition, and the respective residues in ProQ, are highlighted. Right: Enlarged view of the RocC/ProQ 3′ recognition pocket. Residues critical for 3′ recognition are numbered. (**D**) Northern blot analysis to monitor expression of the *rpoS* 3′ fragment in the indicated strains grown in LB at 37°C after induction with 0.02% arabinose for 30 min. A 5′ labeled DNA oligonucleotide specific for the *rpoS* 3′ end was used for detection. tmRNA served as loading control. ProQ expression from the pProQ plasmid in this case includes ProQ fused with dTomato. The levels of ProQ-dTomato are comparable to endogenous ProQ levels [27]. pCtr is the parental plasmid for pProQ and served as an empty vector control. PAPI assay including *in vitro* transcribed *rpoS* 3′ (**E**) or *cspE* 3′ (**F**) fragments with increasing concentrations of ProQ. The ProQ concentrations ranged from 30–480 nM (**E**), and 30–240 nM (**F**).

PNPase-dependent degradation of RNA transcripts carrying an intrinsic terminator occurs through an iterative process, involving cycles of PAPI-mediated polyadenylation and PNPase-dependent degradation from the 3′ end [32]. The dependence of PAPI and PNPase for ProQ-mediated stabilization of the *rpoS* mRNA (Fig. 5) indicated that ProQ directly interferes with this process. If so, it was important to clarify whether ProQ preferentially binds nascent 3′ ends to interfere with PAPI activity, or polyadenylated transcripts to interfere with PNPase activity. To this end, we performed binding assays between ProQ and the *rpoS* 3′ transcript, with or without a twenty-nucleotide long poly(A) tail. Clearly, ProQ has higher affinity for the non-polyadenylated RNA compared to the polyadenylated species (Fig. 6A and B). The structural basis for 3′ recognition by a ProQ/FinO-family protein was recently demonstrated in a crystal structure of protein RocC in complex with its sRNA target RocR [8]. Aligning an Alphafold model of the ProQ/FinO-domain of *S*.Tm ProQ to the RocC/RocR structure suggested structural conservation of critical residues of the 3′ end binding pocket (Fig. 6C). In line with this, mutating one of these residues (mutation R80H) completely abolished ProQ-dependent stabilization of *rpoS-3′*, while a ProQ mutant previously shown to retain RNA-binding activity (A18V [27]) conferred stabilization to the same level as the wild-type protein (Fig. 6D). This indicates that ProQ similar to RocC specifically recognizes 3′ ends, and would predict that ProQ interferes with PAPI-mediated polyadenylation rather than exonuclease activity. To directly test this, we performed *in vitro* polyadenylation reactions. Incubating the *rpoS* 3′ transcript with purified PAPI and ATP resulted in extensive polyadenylation, verifying that the *rpoS* 3′ end is a substrate for PAPI (Fig. 6E). Pre-incubating the *rpoS* 3′ transcript with increasing concentrations of ProQ prior to addition of PAPI resulted in a concentration-dependent reduction of polyadenylation, with almost complete inhibition at ProQ concentrations above 250 nM (Fig. 6E). We have previously shown that ProQ-dependent stabilization of the *cspE* mRNA *in vivo* is dependent on RNase II [5]. Considering that RNase II activity is strongly stimulated by polyadenylation, we repeated the *in vitro* PAPI assay with the *cspE* 3′UTR as substrate. While addition of PAPI and ATP alone conferred efficient polyadenylation of *cspE*, increasing concentrations of ProQ inhibited this activity (Fig. 6F). Together, this indicates that binding of ProQ to nascent RNA 3′ ends counteracts PAPI-mediated polyadenylation, and consequently, exoribonucleolytic degradation.

### ProQ-dependent effects on gene expression require PAPI

**Figure 7.**
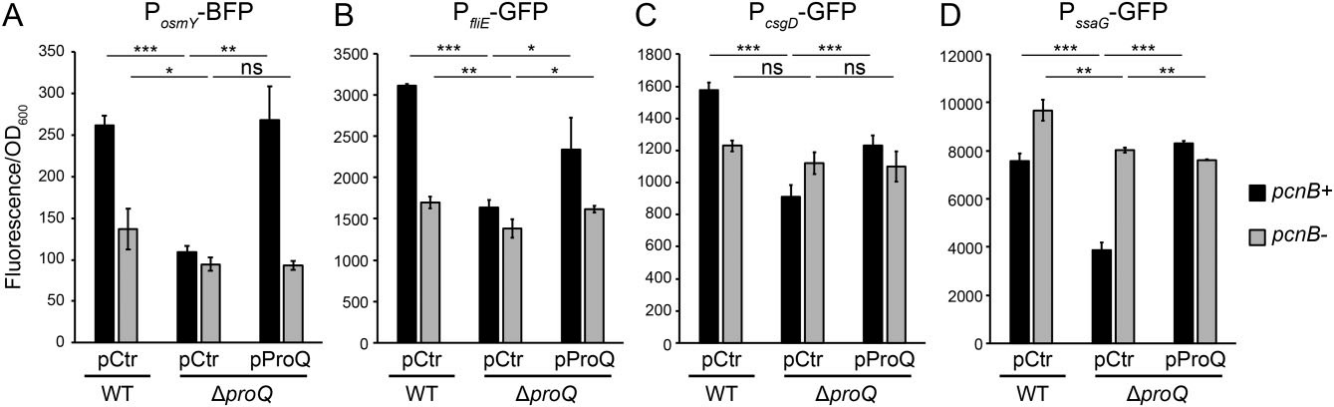
Loss of PAPI abolishes ProQ-dependent expression of virulence-related pathways. Fluorescence from reporter constructs P*_osmY_*-BFP (**A**) and P*_csgD_*-GFP (**C**) was monitored from the indicated strains grown in LB at 28°C. Fluorescence from reporter construct P*_fliE_*-GFP (**B**) and P*_ssaG_*-GFP (**D**) was monitored from the indicated strains grown at 37°C in LB or SPI2-inducing medium, respectively. Background fluorescence has been subtracted. Bars show mean values from three biological replicates. Error bars denote standard deviation. Statistical difference was determined using a two-tailed *t*-test (*P < .1; **P < .05; ***P < .01).

Deletion of *proQ* in *S*.Tm reduces expression of genes involved in several virulence-related traits, including flagellar motility [27], intracellular survival [14, 15], osmotic stress [11], and biofilm (Fig. 1). However, the mechanistic bases for these effects have remained unclear. To test if ProQ-mediated inhibition of PAPI extends beyond *rpoS* and *cspE*, we monitored expression from transcriptional fusions as a proxy for each of these pathways. As expected, deletion of *proQ* resulted in lower expression of each fusion, while complementation with ProQ expressed *in trans* fully or partly restored wild-type expression (Fig. 7). However, deleting *pcnB* strongly reduced ProQ’s effect on expression on each of the tested promoter fusions, suggesting that ProQ-dependent inhibition of PAPI activity may be a general mechanism.

## Discussion

In this study, we address the mechanistic basis for ProQ’s role in biofilm formation. Our results demonstrate that decreased biofilm formation in the absence of ProQ is due to reduced levels of the alternative sigma factor RpoS. This is caused by decreased levels of the *rpoS* mRNA when *proQ* is deleted. Results from genetic and biochemical experiments show that ProQ binds to the nascent *rpoS* 3′ end and inhibits PAPI-mediated polyadenylation and subsequent degradation by PNPase. In the absence of PAPI, previously reported effects on ProQ-dependent gene expression become insensitive to deletion of *proQ*, indicating that inhibition of PAPI activity is a general function of ProQ.

From the collective literature on ProQ/FinO proteins, some common themes are beginning to emerge. First, mapping of ligand binding sites *in vivo*, biochemical characterization of ProQ RNA recognition, large-scale mutagenesis studies, and structural studies of ProQ/FinO proteins alone or in complex with RNA, indicate that the ProQ/FinO domain has evolved to specifically recognize RNA 3′ ends at intrinsic terminators. Here, both the terminator structure and the accessibility of the 3′ end appear to be important for binding. Global ProQ-RNA crosslinking approaches in *S*.Tm, *E. coli*, *Pasteurella multocida*, and *N. meningitidis* showed that ProQ binding sites are typically located at the 3′ end of RNA ligands [3–5, 7, 15]. Biochemical experiments have shown that destabilizing the terminator stem-loop structure, substantially shortening *or* extending the single stranded 3′ end, or changing the 3′ hydroxyl group of the terminal nucleotide to a phosphate group, all impair ProQ/FinO domain binding [5, 8–10]. Recently, a crystal structure of the RocC–RocR complex revealed that the RocR 3′ end is buried in a binding pocket in RocC [8]. The key residues of the binding pocket are among the most highly conserved positions in ProQ/FinO family proteins [8], and were identified as critical residues for binding and regulation in three independent mutagenesis studies [27, 33, 34], suggesting that key features of the RocC–RocR complex at least partly can be extrapolated to ProQ/FinO proteins in general.

Second, transcriptomic studies and global RNA stability measurements [3, 4, 6, 7, 11, 14], as well as steady-state and decay measurements of particular RNA ligands [3, 5, 7, 15, 17, 27, 34–36], repeatedly associate ProQ/FinO proteins with RNA ligand stability. The specific binders FinO and RocC increase the cellular levels of their respective sRNA ligands [36–40], and globally acting ProQ proteins in various bacterial species promote RNA ligand stability, both with respect to sRNAs and mRNAs. However, a mechanistic explanation for how ProQ/FinO proteins promote RNA ligand stability has been lacking. The results presented in this study suggest a general mechanism for ProQ/FinO proteins in stabilizing RNA ligands through preventing PAPI-mediated polyadenylation. The above-mentioned common themes of ProQ/FinO proteins—3′ end recognition and RNA ligand stabilization—are entirely congruent with the mechanism proposed here.

This said, it should be noted that 3′ binding by ProQ/FinO proteins is not always associated with RNA ligand stabilization. In *S*.Tm, deletion of *proQ* leads to destabilization of only a fraction of RNAs harboring a 3′ end ProQ binding site [11]. If ProQ generally prevents polyadenylation and, indirectly, exonucleolytic degradation, why are not all ligands destabilized in the absence of ProQ? The answer likely reflects that 3′ exonucleolytic activity is not the primary factor for degradation of full-length RNAs. The rate-limiting step for the decay of most RNAs in *E. coli*, and most likely *S*.Tm, is RNase E-mediated endonucleolytic cleavage, while the major function of exonucleases is to turn over degradation products generated by this enzyme [12, 41]. Hence, for many full-length RNA transcripts, perturbations of exonucleolytic activity at the 3′ end will be obscured by the dominant activity of RNase E and result in small or no effects when monitoring steady-state levels and/or decay rates. If so, has ProQ evolved to protect 3′ ends from a degrading activity that in many cases does not matter for the overall stability of the bound RNA? This may be connected to the nature of ProQ binding motifs, i.e. intrinsic terminators. Due to their important function in transcription, they may be difficult to evolve so that only a subset of terminators confer ProQ binding. Perhaps there is a sufficiently strong selective pressure to maintain ProQ-dependent stabilization of a specific set of RNA ligands to tolerate non-productive interactions that do not affect RNA ligand stability.

Alternatively, controlling the stability of full-length RNAs may not be the main function of ProQ, but rather a consequence of another process. For instance, ProQ may hinder exoribonuclease-dependent 3′ truncation into open reading frames, which would yield non-stop mRNAs and ribosome stalling. This would be compatible both with inhibition of PAPI activity and the lack of measurable effects on stability for specific RNA ligands. We are currently testing this and other hypotheses to clarify the role(s) of ProQ/FinO proteins.

## Supplementary Material

gkaf103_Supplemental_File

## Data Availability

The data underlying this article are available in the article and in its online supplementary material.

## Notes

Present address: Helmholtz Institute for RNA-based Infection Research (HIRI), Helmholtz Centre for Infection Research (HZI), 97080 Würzburg, Germany
