# Supplementary material for: ProQ prevents mRNA degradation through inhibition of poly(A) polymerase
Source: Nucleic Acids Res. 2025 Feb 27;53(5):gkaf103. doi: 10.1093/nar/gkaf103 (PMC12086684; doi:10.1093/nar/gkaf103)
Supplement: gkaf103_Supplemental_File [file gkaf103_supplemental_file.pdf]

Supplementary material for

ProQ prevents mRNA degradation through inhibition of poly(A) polymerase

Sofia Bergman[1], Christopher Birk[2] and Erik Holmqvist[1*]

Affiliations:

[1] Department of Cell and Molecular Biology, Biomedical Centre, Uppsala University, Uppsala, Sweden

[2] Current address: Helmholtz Centre for Infection Research (HZI), Helmholtz Institute for RNA-based Infection Research (HIRI), Würzburg, Germany.

* To whom correspondence should be addressed.

Erik Holmqvist

Tel: +46 18 471 4073

Email: erik.holmqvist@icm.uu.se

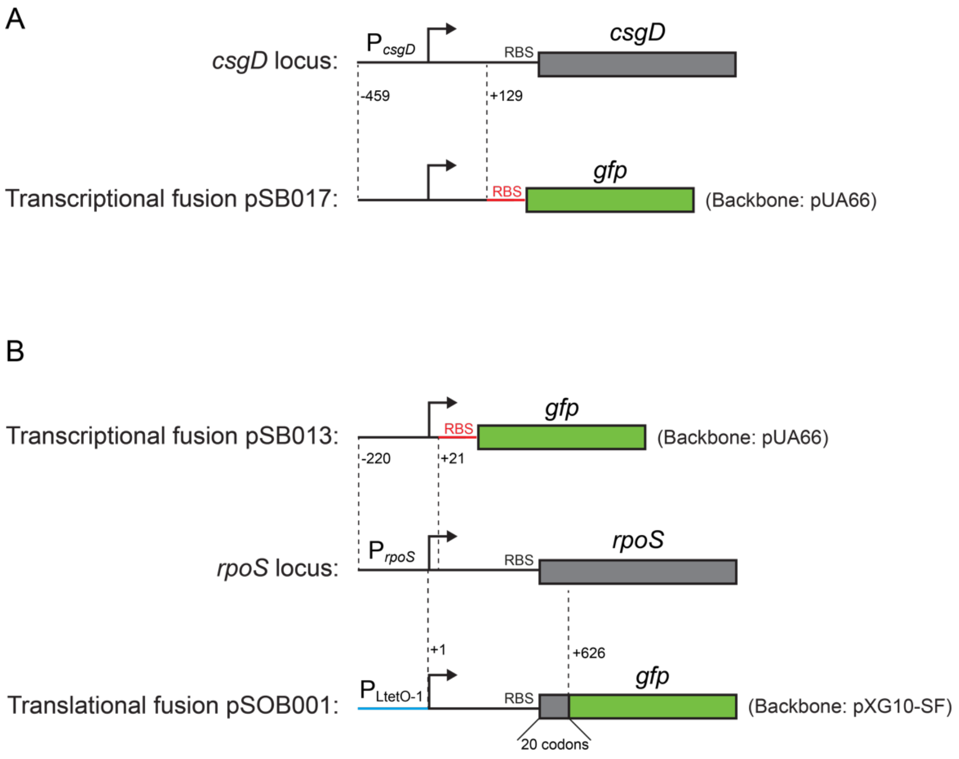

Figure S1. Schematic description of the reporter fusions constructed in this study. (**A**) Construction of the *csgD* promoter fusion pSB017. The insert spans from 459 nt upstream to 129 nt downstream relative to the *csgD* transcriptional start site (TSS) (indicated with an arrow). (**B**) Transcriptional (pSB013) and translational (pSOB001) *rpoS* fusions. The insert in pSB013 spans from 220 nt upstream to 21 nt downstream relative to the *rpoS* TSS. The insert in pSOB001 spans from the *rpoS* TSS to 626 nt downstream of the TSS, thereby including 20 codons of the CDS.

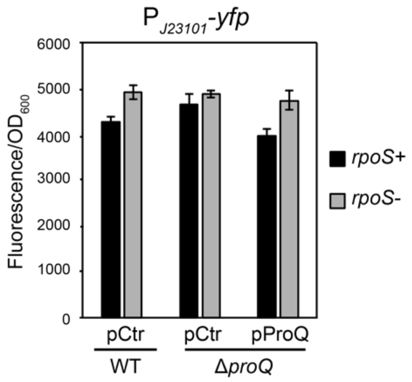

Figure S2. Measurements of YFP expression from the synthetic RpoD-dependent promoter J23101 in WT, Δ*proQ*, Δ*rpoS* and Δ*proQ*Δ*rpoS* strains during growth in LB medium. ProQ expression from the pProQ plasmid is comparable to endogenous ProQ levels. pCtr is the parental plasmid for pProQ and served as an empty vector control. Bars show mean values from three biological replicates. Error bars show standard deviation.

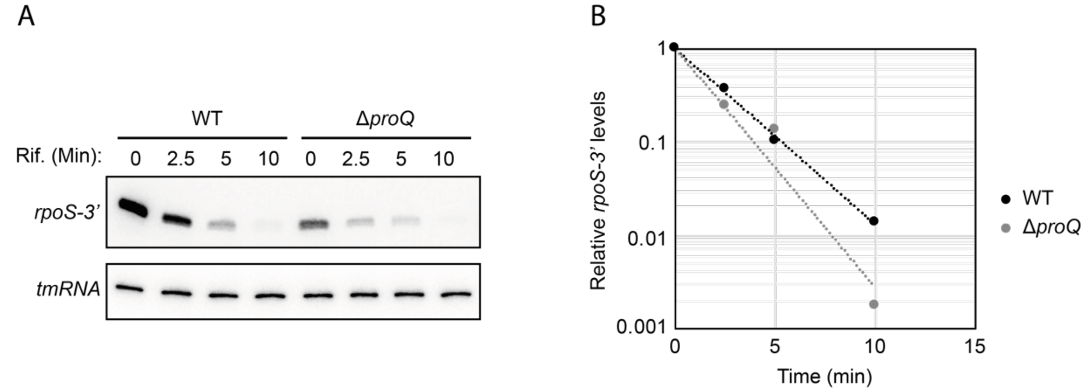

Figure S3. (**A**) Rifampicin experiment monitoring the decay of the *rpoS* 3' fragment in the indicated strains. Strains were grown in LB to an OD_{600} of 2.0, and expression of the *rpoS* 3' fragment was induced for 30 min by addition of 0.002% arabinose. To stop transcription, rifampicin (final concentration of 500 µg/ml) was added and samples were taken at indicated timepoints. The extracted RNA was separated on a polyacrylamide gel and transferred to a membrane. A 5'-labeled DNA oligonucleotide specific for the *rpoS* 3' end (see Table S2) was used for detection. tmRNA served as loading control. (**B**) Quantification of the membrane shown in A. The *rpoS*-3' band intensities were normalized to tmRNA and the values are displayed relative to the 0 time point for WT (black circles) and Δ*proQ* (grey circles), respectively.

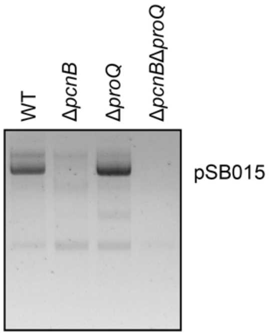

Figure S4. Comparison of pSB015 copy number in the indicated strains. The strains were grown in LB overnight, and an equal amount of bacteria was used for plasmid extraction from each strain (GeneJET plasmid miniprep kit). Equal volumes of isolated plasmids from each cultured were separated on a 0.7% agarose TAE (40 mM Tris, 20 mM acetic acid, 1 mM EDTA) gel with SYBR-Safe.

Table S1. Bacterial strains used in this study

Strain number	Strain background	Relevant genotype	Selection marker	Reference
JVS-1574	SL1344			(1)
JVS-11364	SL1344	*ΔproQ:Km*	KmR	(2)
JVS-11365	SL1344	*ΔproQ*		(2)
3760, McClelland collection	14028s	*ΔrpoS*	KmR	(3)
EHS-2506	SL1344	*ΔrpoS::Km*	KmR	This study
EHS-2588	SL1344	*ΔrpoS*		This study
EHS-2626	SL1344	*ΔrpoSΔproQ::Km*	KmR	This study
EHS-2646	SL1344	*ΔrpoSΔproQ*		This study
EHS-1289	SL1344	*ΔpcnBΔproQ::Km*	KmR	(4)
EHS-4347	SL1344	*ΔpcnBΔproQ*		This study
EHS-1515, JVS-871	SL1344	*Δrnr*		(4)
EHS-1516, JVS-872	SL1344	*Δpnp*		(4)
EHS-1517, JVS-873	SL1344	*Δrnb*		(4)
EHS-1518, JVS-869	SL1344	*ΔpcnB*		(4)
EHS-1286	SL1344	*ΔrnrΔproQ::Km*	KmR	(4)
EHS-1287	SL1344	*ΔpnpΔproQ::Km*	KmR	(4)
EHS-1288	SL1344	*ΔrnbΔproQ::Km*	KmR	(4)

Table S2. Oligonucleotides used in this study

Oligo number	Sequence (5' -> 3')	Description
EHO-1902	GTTTTTctcgagGAACGATTTCCGCGATCTGG	Cloning pSB013
EHO-1903	GTTTTTggatccGTTAGCACTTTGTTCACCTGA	Cloning pSB013
EHO-2328	GTTTTTctcgagAAGCTCATACCAAAGTGCTA	Cloning pSB017
EHO-2329	GTTTTTggatccTTAATCGCACATCTGACAGC	Cloning pSB017
EHO-1884	GTTTTATGCATTCAGGTGAACAAAGTGCTAAC	Cloning pSOB001
EHO-1885	GTTTTGCTAGCGTTCTCATCAAATTCCGCG	Cloning pSOB001
EHO-1945	GTTTTGCTAGCCGCGAAATTCTGCAGACG	Cloning pSB015
EHO-1946	GTTTTAAGCTTCCAATGGTGCCGAGTATC	Cloning pSB015
EHO-1958	TGTAGGCCGTGAAATCGGTC	qRT-PCR, *rpoS*
EHO-1959	CCTGCGTCTGCAGAATTTCG	qRT-PCR, *rpoS*
EHO-2368	CGCAAACGACGAAACCTACG	qRT-PCR, tmRNA
EHO-2369	TGACCTCTCTTGATCCCCGT	qRT-PCR, tmRNA
EHO-2311	ACTTATAGCTTCGCGACAAGGCGCTCATTCAT GGGAACAG	Northern blot probe for SL1344 *rpoS*
EHO-0867	TGGTGGAGCTGGCGGGAGTT	Northern blot probe for SL1344 tmRNA
EHO-2366	gtttttttaatacgactcactataggGCGAAATTCTGCAGA CGCAGGGGCTGAATATCGAAGCGCTGTTCCG	*rpoS* 3´ DNA template for *in vitro* transcription
EHO-2367	AAAAAAAAGGCCAGTCGACAGACTGGCCTTTT TTTGACAAGGGTACTTACTCGCGGAACAGCG CTTCGATATT	*rpoS* 3´ DNA template for *in vitro* transcription
EHO-2464	gtttttttaatacgactcactataggGCGAAATTCTGCAGA CGCAGGGGCTGAATATCGAAGCGCTGTTCCG CGAGTAAGTA	*rpoS* 3´-A20 DNA template for *in vitro* transcription
EHO-2465	TTTTTTTTTTTTTTTTTTTTAAAAAAAAGGCCAG TCGACAGACTGGCCTTTTTTTGACAAGGGTAC TTACTCGCGGAACAGCG	*rpoS* 3´-A20 DNA template for *in vitro* transcription
EHO-0845	gtttttttaatacgactcactataggGCTGCAAACGTAACT GCTCTGtaaGCATACGTCCGACAGCAAGATTT CAAAACCCGCCCTTTCGGCGGGTTTTTTTT	*cspE* DNA template for *in vitro* transcription
EHO-0846	AAAAAAAACCCGCCGAAAGGGCGGGTTTTGA AATCTTGCTGTCGGACGTATGCttaCAGAGCAG TTACGTTTGCAGCCCTATAGTGAGTCGTATTA AAAAAAAC	*cspE* DNA template for *in vitro* transcription

Table S3. Plasmids used in this study

Plasmid	Description	Parental plasmid	Selection marker	Reference
pAR007	Empty vector; PLlacO-C promoter and LacI repressor		TetR	(5)
pAR009	IPTG-inducible ProQ	pAR007	TetR	(5)
pXG-1	Empty vector; PLtetO-1 promoter		CmR	(6)
pXG10-SF	Empty vector; PLtetO-1 promoter and *gfp*		CmR	(7)
pSOB001	Translational fusion *rpoS*	pXG10-SF	CmR	This study
pUA66	Empty vector, promotorless; *gfp* gene		KmR	(8)
pYMB005	Transcriptional fusion P*ssaG-gfp*	pUA66	KmR	(9)
pSB017	Transcriptional fusion P*csgD-gfp*	pUA66	KmR	This study
pAR022	Transcriptional fusion P*fliE-gfp*	pUA66	KmR	(5)
pSB013	Transcriptional fusion P*rpoS-gfp*	pUA66	KmR	This study
pEH809	Empty vector, P*araBAD* promoter	pBR322	CmR	(9)
pSB015	Expression of *rpoS* 3' fragment	pEH809	CmR	This study
pSK2938	Transcriptional fusion P*osmY-bfp* and PJ23101-*yfp*		KmR	(10)
pSC101	Empty vector, promotorless; *bfp* gene		KmR	(10)
pCP20	Remove resistant cassette		CmR	(11)
pAR013	IPTG-inducible dTomato		TetR	(5)
pAR018	IPTG-inducible dTomato-ProQ		TetR	(5)
pAR029	*proQ* A18V mutation introduced to pAR018		TetR	(5)
pAR033	*proQ* R80H mutation introduced to pAR018		TetR	(5)

REFERENCES SUPPLEMENTARY

1. Hoiseth,S.K. and Stocker,B.A.D. (1981) Aromatic-dependent Salmonella typhimurium are non-virulent and effective as live vaccines. *Nature*, 291, 238–239.

2. Smirnov,A., Förstner,K.U., Holmqvist,E., Otto,A., Günster,R., Becher,D., Reinhardt,R. and Vogel,J. (2016) Grad-seq guides the discovery of ProQ as a major small RNA-binding protein. *Proc Natl Acad Sci U S A*, 113, 11591–11596.

3. Porwollik,S., Santiviago,C.A., Cheng,P., Long,F., Desai,P., Fredlund,J., Srikumar,S., Silva,C.A., Chu,W., Chen,X., *et al.* (2014) Defined single-gene and multi-gene deletion mutant collections in salmonella enterica sv typhimurium. *PLoS One*, 9, e99820.

4. Holmqvist,E., Li,L., Bischler,T., Barquist,L. and Vogel,J. (2018) Global Maps of ProQ Binding In Vivo Reveal Target Recognition via RNA Structure and Stability Control at mRNA 3′ Ends. *Mol Cell*, 70, 971-982.e6.

5. Rizvanovic,A., Kjellin,J., Söderbom,F. and Holmqvist,E. (2021) Saturation mutagenesis charts the functional landscape of Salmonella ProQ and reveals a gene regulatory function of its C-terminal domain. *Nucleic Acids Res*, 49, 9992–10006.

6. Urban,J.H. and Vogel,J. (2007) Translational control and target recognition by Escherichia coli small RNAs in vivo. *Nucleic Acids Res*, 35, 1018–1037.

7. Corcoran,C.P., Podkaminski,D., Papenfort,K., Urban,J.H., Hinton,J.C.D. and Vogel,J. (2012) Superfolder GFP reporters validate diverse new mRNA targets of the classic porin regulator, MicF RNA. *Mol Microbiol*, 84, 428–445.

8. Zaslaver,A., Bren,A., Ronen,M., Itzkovitz,S., Kikoin,I., Shavit,S., Liebermeister,W., Surette,M.G. and Alon,U. (2006) A comprehensive library of fluorescent transcriptional reporters for Escherichia coli. *Nat Methods*, 3, 623–628.

9. Bergman,S., Andresen,L., Kjellin,J., Martinez Burgo,Y., Geiser,P., Baars,S., Söderbom,F., Sellin,M.E. and Holmqvist,E. (2024) ProQ-dependent activation of Salmonella virulence genes mediated by post-transcriptional control of PhoP synthesis . *mSphere*, 9.

10. Virtanen,P., Wäneskog,M. and Koskiniemi,S. (2019) Class II contact-dependent growth inhibition (CDI) systems allow for broad-range cross-species toxin delivery within the Enterobacteriaceae family. *Mol Microbiol*, 111, 1109–1125.

11. Cherepanov,P.P. and Wackernagel,W. (1995) Gene disruption in Escherichia coli: TcR and KmR cassettes with the option of Flp-catalyzed excision of the antibiotic-resistance determinant. *Gene*, 158, 9–14.
